# Performance evaluation of SARS-CoV-2 rapid diagnostic tests in Nigeria: A cross-sectional study

**DOI:** 10.1371/journal.pgph.0003371

**Published:** 2024-07-15

**Authors:** Akhere A. Omonkhua, Adedayo Faneye, Kazeem S. Akinwande, Okpokoro Evaezi, Nathan Y. Shehu, Adedeji Onayade, Chinwe Lucia Ochu, Mustapha Popoola, Nnadi Emmanuel, Temitope Ojo, Cornelius Ohonsi, Abdullahi Abubakar, Elizabeth Odeh, Paul Akinduti, Onikepe Folarin, John Samson Bimba, Ehimario Igumbor, Kelly Elimian, Victory Fabian Edem, Luka Pam D., Tunde Olusola, Loretta Ntoimo, Michael Olugbile, Adewale Victor Opayele, Ibrahim Kida, Shwe David, Augustine Onyeaghala, Isaac Igbarumah, Omosivie Maduka, Magaji A. Mahmoud, Abdul Rahman El-Fulatty, David O. Olaleye, Omale Simon, Iriagbonse Iyabo Osaigbovo, Darlington Ewaen Obaseki, Afolaranmi Tolulupe, Christian Happi, Yusuf Bara Jibrin, Friday Okonofua, Timan Eliya, Gomerep Simji, Izang, Joy Abi, Emmanuel Ameh, Ibrahim Mahmood Maigari, Sulaiman Alhaji, Ifedayo Adetifa, Babatunde Salako, Suleiman Bogoro, Chikwe Ihekweazu, Georgina N. Odaibo, COVID-19 RDT Validation in Nigeria (CORVAN) study group

**Affiliations:** 1 Centre of Excellence in Reproductive Health Innovation (CERHI), University of Benin, Benin City, Nigeria; 2 Department of Medical Biochemistry, University of Benin, Benin City, Nigeria; 3 Department of Virology, College of Medicine, University of Ibadan, Ibadan, Nigeria; 4 Department of Chemical Pathology and Immunology, Federal Medical Centre Abeokuta, Abeokuta, Nigeria; 5 International Research Centre of Excellence, Institute of Human Virology, Abuja, Nigeria; 6 West African Center for Emerging Infectious Diseases (WAC-EID), Jos University Teaching Hospital, Jos, Nigeria; 7 Institute of Public Health, Obafemi Awolowo University, Ile-Ife, Nigeria; 8 Nigeria Centre for Disease Control & Prevention, Abuja Nigeria; 9 Nigeria COVID-19 Research Coalition, Abuja, Nigeria; 10 Tertiary Education Trust Fund, Abuja, Nigeria; 11 Plateau State University, Bokkos, Plateau State, Nigeria; 12 Federal University Teaching Hospital, Abakiliki, Ebonyi State, Nigeria; 13 Department of Microbiology, Covenant University, Ota, Ogun State, Nigeria; 14 African Centre of Excellence for Genomics of Infectious Diseases, Redeemers University, Ede, Nigeria; 15 Zankli Research Centre, Bingham University, Karu, Nigeria; 16 Centre for Infectious Disease Research, Nigerian Institute of Medical Research, Lagos, Nigeria; 17 School of Health Systems and Public Health, University of Pretoria, Pretoria, South Africa; 18 Department of Public Health, Walter Sisulu University, Mthatha, South Africa; 19 Department of Microbiology, Faculty of Life Sciences, University of Benin, Benin City, Edo State, Nigeria; 20 Department of Immunology, University of Ibadan, Ibadan, Nigeria; 21 National Veterinary Research Institute (NVRI), Vom, Plateau State, Nigeria; 22 Department of Demography and Social Statistics, Federal University, Oye-Ekiti, Ekiti State, Nigeria; 23 The World, Bank, Abuja, Nigeria; 24 University of Maiduguri Teaching Hospital, Maiduguri, Borno State, Nigeria; 25 Department of Chemical Pathology, University College Hospital, Ibadan, Nigeria; 26 Molecular Virology Laboratory, University of Benin Teaching Hospital, Benin City, Nigeria; 27 University of Port Harcourt, Port Harcourt, Rivers State, Nigeria; 28 Faculty of Science, Ahmadu Bello University, Zaria, Nigeria; 29 University of Jos, Jos, Plateau State, Nigeria; 30 Department of Medical Microbiology, School of Medicine, College of Medical Sciences, University of Benin, Benin City, Nigeria; 31 Office of the Chief Medical Director, University of Benin Teaching Hospital, Benin City, Nigeria; 32 Abubakar Tafawa Balewa University Teaching Hospital (ATBUTH), Bauchi, Nigeria; 33 Department of Obstetrics and Gynaecology, University of Benin, Benin City, Nigeria; 34 Nigerian Institute of Medical Research, Lagos, Nigeria; 35 World Health Organization Hub for Pandemic and Epidemic Intelligence, Berlin, Germany; UMass Chan Medical School - Baystate Medical Center, UNITED STATES OF AMERICA

## Abstract

The COVID-19 pandemic challenged health systems globally. Reverse transcription polymerase chain reaction (RT-PCR) is the gold standard for detecting the presence of SARS-CoV-2 in clinical samples. Rapid diagnostic test (RDT) kits for COVID-19 have been widely used in Nigeria. This has greatly improved test turnover rates and significantly decreased the high technical demands of RT-PCR. However, there is currently no nationally representative evaluation of the performance characteristics and reliability of these kits. This study assessed the sensitivity, specificity, and predictive values of ten RDT kits used for COVID-19 testing in Nigeria. This large multi-centred cross-sectional study was conducted across the 6 geo-political zones of Nigeria over four months. Ten antigen (Ag) and antibody (Ab) RDT kits were evaluated, and the results were compared with RT-PCR. One thousand, three hundred and ten (1,310) consenting adults comprising 767 (58.5%) males and 543 (41.5%) females participated in the study. The highest proportion, 757 (57.7%), were in the 20–39 years’ age group. In terms of diagnostic performance, Lumira Dx (61.4, 95% CI: 52.4–69.9) had the highest sensitivity while MP SARS and Panbio (98.5, 95% CI: 96.6–99.5) had the highest specificity. For predictive values, Panbio (90.7, 95% CI: 79.7–96.9) and Lumira Dx (81.2, 95% CI: 75.9–85.7) recorded the highest PPV and NPV respectively. Ag-RDTs had better performance characteristics compared with Ab-RDTs; however, the sensitivities of all RDTs in this study were generally low. The relatively high specificity of Ag-RDTs makes them useful for the diagnosis of infection in COVID-19 suspected cases where positive RDT may not require confirmation by molecular testing. There is therefore the need to develop RDTs in-country that will take into consideration the unique environmental factors, interactions with other infectious agents, and strains of the virus circulating locally. This may enhance the precision of rapid and accurate diagnosis of COVID-19 in Nigeria.

## Introduction

Coronavirus disease 2019 (COVID-19), which was declared a pandemic by the World Health Organisation (WHO) in March 2020, is a crisis that has challenged health systems globally. Over 774 million infections and 7 million deaths have been documented worldwide as of January 14, 2024, with about 9.60 million cases occurring in Africa [1]. Early and rapid diagnosis is crucial for rapidly identifying and isolating infected individuals in a bid to slow down transmission, provide timely clinical management to those affected, and protect health systems operations through triaging at admissions [2].

Reverse transcription polymerase chain reaction (RT-PCR), a very sensitive and specific technique, is the gold standard testing method for detecting the presence of SARS-CoV-2 in clinical samples [3]. However, it is resource-intensive requiring huge investments, including highly skilled manpower, expensive machines, elaborate specimen transport systems, and costly reagents and consumables [4]. More importantly, RT-PCR techniques cannot be implemented in remote settings in Africa, including Nigeria, due to a lack of appropriate infrastructure [2, 5].

With the frequent emergence of variants of the virus, which have fuelled new outbreaks of the pandemic [6], coupled with low vaccine uptake and coverage [7], the world is faced with a looming possibility that COVID-19 will persist, transitioning from a pandemic to an endemic disease [8]. Diagnostic platforms that are sustainable for the long haul are necessary, to maintain widespread accessibility and inform public health decision-making. Rapid diagnostic tests (RDTs), such as antibody (Ab) and antigen (Ag) RDTs, are attractive alternatives to the technically demanding, resource intensive, though highly sensitive molecular platforms for several economic, public health, and operational reasons [5]. For example, from the economic perspective, RDTs are cheaper to produce and mostly do not require power to store reagents and conduct tests. The World Health Organization recommends deploying rapid tests with a sensitivity of at least 80% and specificity greater than 97% compared to the RT-PCR in applicable settings [9]. Currently, the diagnostic performance of most RDTs in the market is yet to be adequately evaluated in field situations in many African settings. Despite the near-universal roll-out of these tests, real-world data remains limited to evaluations conducted in Kenya [10], Uganda [11], South Africa [12], Cameroon [13], and Ghana [14]. Since the country’s third wave of COVID-19 in July of 2021, WHO and the Nigeria Centre for Disease Control (NCDC) recommended kits have been deployed to scale up testing in Nigeria. However, their performance and those of other commercially available kits are yet to be evaluated in a nationally representative field study to ascertain their reliability. The role of accurate and reliable diagnosis in the management and control of an infection cannot be overemphasised. This prospective study was therefore conducted to assess the sensitivity, specificity, and predictive values of RDTs available for point-of-care COVID-19 diagnosis in Nigeria.

## Materials and methods

### Study area/design

Seven teams, coordinated by the Nigeria COVID-19 Research Coalition (NCRC), designed a harmonised protocol to evaluate the performance characteristics of available antigen and antibody SARS-CoV-2 rapid diagnostic kits (RDTs) in Nigeria. This multi-centre cross-sectional study was conducted in thirteen (13) states and the Federal Capital Territory (FCT), within the six (6) geo-political zones of Nigeria (Table 1). It was carried out for four months (October 2021 to January 2022) and spanned through two successive waves (3^rd^ and 4^th^) of the COVID-19 pandemic. Government-approved testing centres were used to collect samples from consenting participants suspected of COVID-19.

**Table 1:** Study sites for the validation of SARS CoV-2 rapid diagnostic kits in Nigeria.

S/No	Geo-political Zone	State
1	South-West	Ogun, Osun, Oyo
2	South-East	Ebonyi
3	South-South	Edo, Rivers
4	North-West	Bauchi, Borno
5	North-East	Jigawa, Kaduna
6	North-Central	Abuja, Nassarawa, Plateau

A total of six antigen RDT kits were evaluated, including—Lumira Dx SARS-CoV-2 Ag, Abbot Panbio COVID-19 Ag, Lifotronic SARS-CoV-2 Ag, MP Rapid SARS-CoV-2 Ag Card, Mologic COVID-19 Ag Rapid Diagnostic Test, and SGTi-flex COVID-19 Ag. Four (4) antibody RDT kits were also evaluated (SGTi-flex COVID-19 IgM/IgG, SD Biosensor COVID-19 IgM/IgG Combo, RightSign COVID-19 IgG/IgM Rapid Test Cassette, and Genuri Novel Coronavirus (2019-nCoV) IgG/IgM test kit) (Table 2). This list was based on the National Agency for Food and Drug Administration and Control (NAFDAC) recommendation.

**Table 2:** List of COVID-19 rapid diagnostic test kits evaluated.

S/No	Test Kit	Manufacturer	Performance Characteristics*		Target Antigen/antibody
			Sensitivity/Positive Percent Agreement (95%CI)	Specificity/Negative Percent Agreement	
1	Lumira Dx SARS-CoV-2 Ag	Lumira Dx, UK Limited, UK	97.6%	96.6%	Nucleocapsid protein
2	Abbot Panbio COVID-19 Ag	Abbot Rapid Diagnostics, Jena, Germany	91.4% (85.5%- 95.5%)	99.8% (98.8%- 100%)	Nucleocapsid protein
3	Lifotronic SARS-CoV-2 Antigen*	Shenzen Lifotronic Technology Co. Ltd, Guandong Province, China	10/10	10/10	Nucleocapsid protein
4	MP Rapid SARS-CoV-2 Antigen Card	MP Biomedicals, Germany	96.49% (93.11%-99.87%)	99.07% (98.26%-99.98%)	Nucleocapsid protein
5	Mologic COVID-19 Antigen Rapid Diagnostic Test	Mologic Limited, Bedford, UK	85%	98%	Not specified
6	SGTi-flex COVID-19 Ag	Sungentech Inc., Chungcheongbukdo, Republic of Korea	95.06% (90.56%- 97.48%)	99.29% (96.29%- 99.87%)	Nucleocapsid protein
7	SGTi-flex COVID-19 IgM/IgG	Sungentech Inc., Chungcheongbukdo, Republic of Korea	IgM 93.3% (78.7% -98.2%) IgG 93.3% (78.7% -98.2%) Combined IgM/IgG 100% (88.7%-100%)	IgM 90.0% (81.5% -94.8%) IgG 100% (95.4%-100%) Combined IgM/IgG 90.0% (81.5%-94.8%)	Not specified
8	SD Biosensor COVID-19 IgM/IgG Combo	SD Biosensor, Republic of Korea	<7days 69.05% (52.91%-82.38%) ≫7days 94.51% (89.84%- 97.46%) 7–14 days 89.39% (79.36%-95.63%) >14 days 96.94% (91.31%-99.36%)	95.74% (92.31%- 97.94%)	Antibody to Nucleocapsid protein
9	RightSign COVID-19 IgG/IgM Rapid Test Cassette (Biotest RightSign)	Hangzhou Biotest Biotech Company Ltd., Hangzhou, China	91.4% (82.3%-96.8%)	100% (74.1%-100%)	Not specified
10.	Genuri Novel Coronavirus (2019-nCoV) IgG/IgM test kit	Genuri Biotech Inc., Shenzen, China	10/10	20/20	Not Specified

*As indicated by kit manufacturers

### Study participants

**Fig 1:**
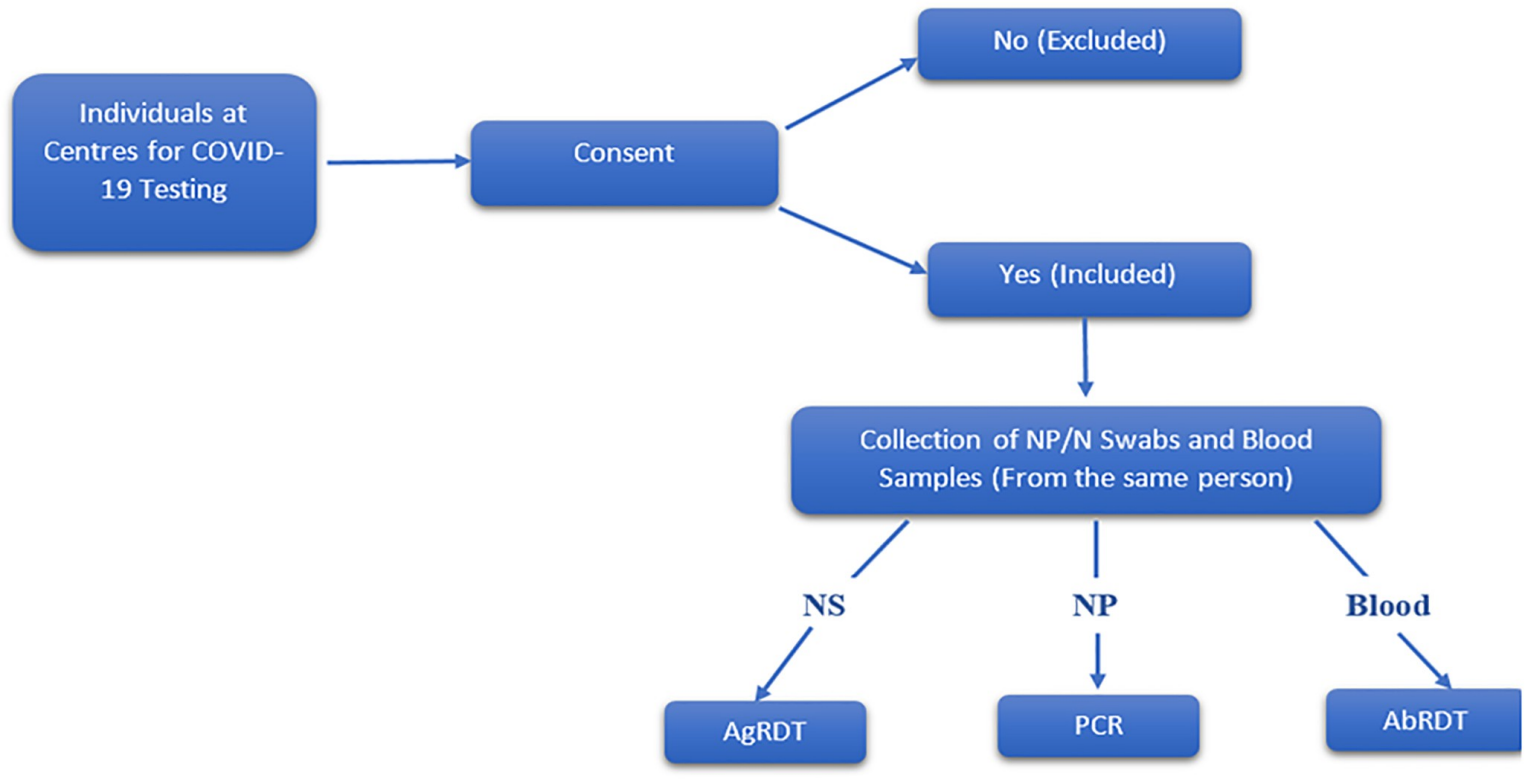
Flowchart for inclusion and exclusion criteria. *NS—Nasal swab; NP–Nasopharyngeal swab.

Consecutive consenting adults (18 years and above) who presented at the various centres for COVID-19 testing were enrolled in the study over four months (October 2021 to January 2022). All sites followed the same inclusion and exclusion criteria as shown in the flowchart in Fig 1.

### Sample size determination

The sample size for the study was determined using RT-PCR as a reference test and the following assumptions: a pre-determined sensitivity value of 70% and specificity value of 99%; the positivity rate of COVID-19 among suspected cases in Nigeria of 10%; maximum marginal error of 5%; and an alpha level of 0.05. The sample size for the study was determined using the formula [15–17]

$$\text{n}=\frac{{\left[{\text{Z}}_{\alpha }\surd 2\;\text{X}\;{\text{P}}^{-}(1-{\text{P}}^{-})+{\text{Z}}_{\beta }\surd {\text{P}}_{1}(1-{\text{P}}_{1})+{\text{P}}_{2}(1-{\text{P}}_{2})\right]}^{2}}{{\left({\text{P}}_{1}-{\text{P}}_{2}\right)}^{2}}$$

where P1 and P2 denote the sensitivity of two alternative point-of-care diagnostic tests respectively for testing the hypothesis. P⁻ is the average of P1 and P2, Zα is the standard normal deviation at a 95% confidence interval (1.96), and Zβ is the statistical power to detect a difference of 10%. Based on the report of a previous study [18], P1 was 88.7% (sensitivity) and 90.6% (specificity) using IgM/IgG based diagnostic test. The calculated sample sizes were 130 and 96 test per point-of-care diagnostic test for each of the 7 study sites based on the sensitivity and specificity assumptions respectively. A minimum sample size of 130 tests per point-of-care diagnostic test for each of the 7 study sites, which gave a total minimum sample size of 910 was adopted.

### Sample collection and processing

**Fig 2:**
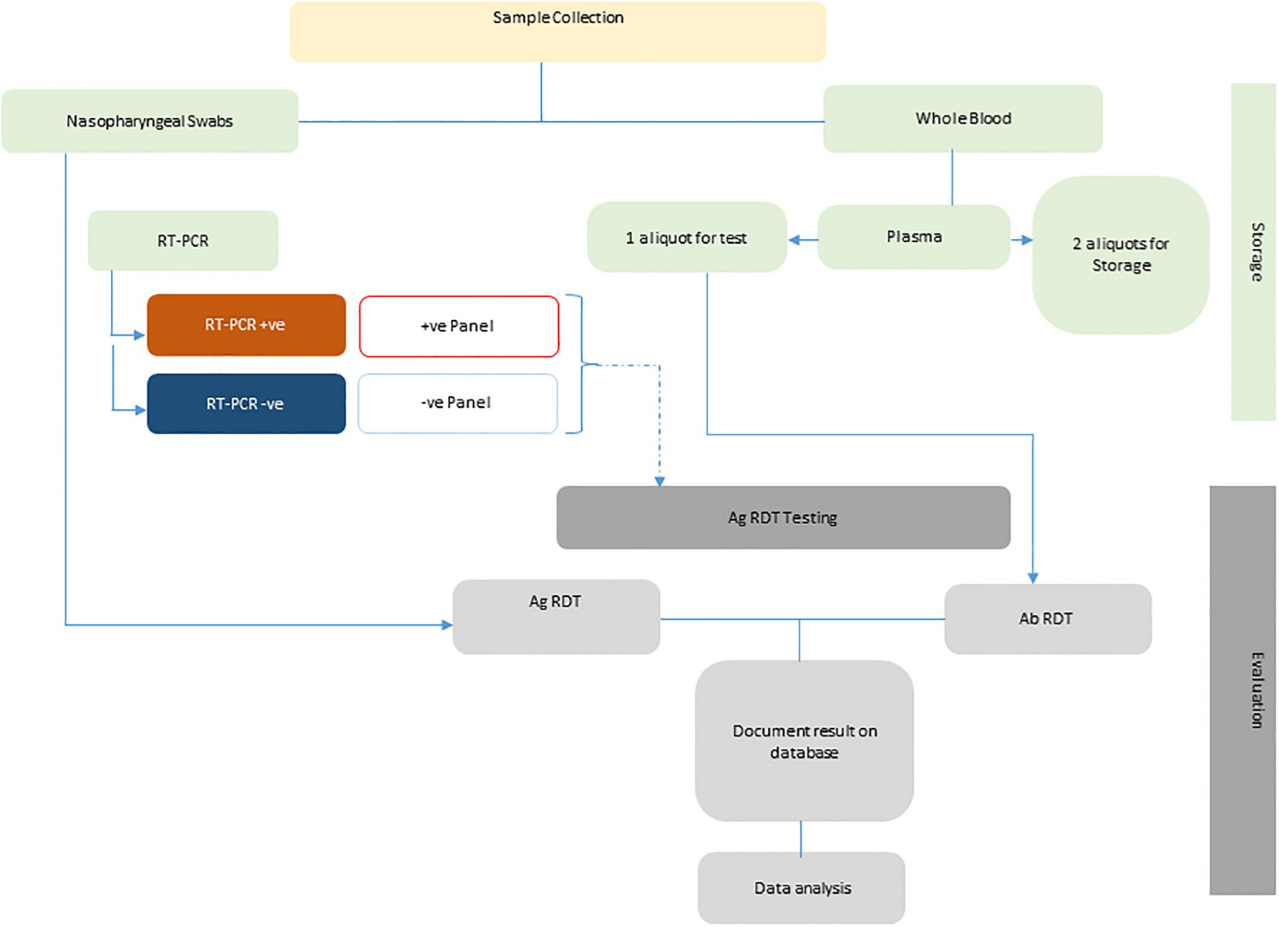
Evaluation process for the validation of SARS-COV-2 rapid diagnostic tests in Nigeria. * RT-PCR—Reverse transcription polymerase chain reaction, RDT—Rapid diagnostic test, Ab–Antibody; Ag–Antigen.

Concomitant nasopharyngeal, oropharyngeal, and nasal swabs as well as whole blood samples, were collected from each consenting individual attending the various sample collection centres for COVID-19 testing. Samples were collected following strict infection prevention and control protocols as outlined in the NCDC specimen collection guidelines. The nasopharyngeal and oropharyngeal swab samples were collected and placed into a viral transport medium (VTM) and stored at 2–7°C before transportation to the molecular laboratory for RT-PCR testing. The nasal swabs were collected and placed in a diluent supplied by the manufacturer of the respective antigen test kits. In addition, 10 ml of blood was collected from each participant into EDTA or plain collection tubes. Samples were transported in cold-box containing ice packs to the laboratories, where they were centrifuged at 3500 rpm for 15 minutes. The plasma or serum was separated and then tested for the presence of SARS-CoV-2 antibodies using RDT kits according to manufacturers’ instructions. Thereafter, aliquots of the samples were shipped to biorepositories for storage and future SARS-CoV-2-related studies. The schema of sample collection and testing is shown in Fig 2.

### Reference method

RT-PCR was used as the gold standard. Viral RNAs were extracted from the naso-/oro-pharyngeal swabs using approved kits supplied by NCDC (the kit supplied was based on what was available at the time) and following the manufacturer’s instructions. Table 3 shows the list of PCR reagents supplied by NCDC during this period. The presence of SARS-CoV-2 in each sample was detected using commercially available RT-PCR kits targeting at least three genes (RdRp, N, ORF1b, and E genes) of the virus. The samples were analysed on a real-time PCR machine using the cycling conditions recommended by the respective kit’s manufacturer. The human housekeeping gene RNAse P was targeted as the internal control for both nucleic acid extraction and PCR for the normalisation of cycle threshold (Ct) values. The Ct values of < 40 were considered positive while values ≥ 40 were considered negative according to the manufacturer’s instructions.

**Table 3:** List of PCR reagents supplied by NCDC.

S/No.	Kit Name	Manufacturer	Target genes
1	Genefinder COVID-19 Plus Real-Amp	Osang Healthcare	N gene, E gene, RDRP
2	Gensig COVID-19 Real-Time PCR Assay	Primerdesign	ORF1ab
3	TaqPath COVID-19 Kit	Thermo Scientific	N Gene, S Gene, ORF1
4	BGI Real-Time Flouresscent RT-PCR kit for Detecting SARS-CoV-2	BGI Genomics	ORF1 ab

### RDT methods

A total of six (6) Ag and four (4) Ab RDT kits were evaluated in this study; all kits were tested in all study sites. All Ag RDTs were lateral flow immunochromatographic tests (LFT) except the Lumira Dx SARS-CoV-2 Ag test (Lumira Dx UK), which is a fluorescent immunoassay (FIA) with a digital machine for detecting and reading the results. All the six Ab RDTs were LFTs capable of detecting and differentiating IgM and IgG. A list of RDTs evaluated, including the target antigen/antibody is found in Table 2. All samples were blindly tested by two different experienced laboratory scientists. The tests were carried out and interpreted according to the manufacturers’ instructions. Nasal swab samples collected for the study were tested for the presence of SARS-CoV-2 antigen while plasma or serum samples were tested for the presence of SARS-CoV-2 antibodies (IgG and IgM) using the various antigen and antibodies detection RDTs respectively.

### Data collection and analysis

Individuals who consented were enrolled in the study; data from all centres were pooled together and statistical analysis was performed for all participants with complete information. Socio-demographic information obtained from study participants and the laboratory results were entered into the research electronic data capture (REDCap) tool. Point estimates of sensitivity, specificity, positive predictive values, negative predictive values, and the area under the receiver operating characteristic curve was used to determine the general performance and characteristics of the RDTs. Participants with indeterminate results on either RDT or PCR reference tests as well as those with missing data were excluded from the final analysis.

Statistical analysis was done using StataCorp. 2021. *Stata Statistical Software*: *Release 17*. College Station, TX: StataCorp LLC. The area under the receiver operating characteristic curve (AUROC) was computed for individual test kits using the sensitivity and 1-specificity of each test to plot a curve across varying cut-off values [19]. AUROC with values > 0.70 were adjudged to possess good to excellent diagnostic capacity, whereas AUROC values < 0.5–0.70 were adjudged to have poor diagnostic accuracy. AUROC values less than 0.5 imply extremely poor discriminative ability [20, 21].

The agreed criteria for percentage concordance were set at 95% sensitivity (positive concordance) and 90% specificity (negative concordance) when compared to the RT-PCR gold standard. We handled missing data using the missing indicator approach.

### Ethical consideration

The National Health Research Ethics Committee (NHREC) approved the validation protocol and all procedures before the initiation of this study (NHREC/01/01/2007-14/12/2020). All procedures followed the harmonized protocol for the study and were in line with the WHO Research and Development priorities and blueprint for the novel coronavirus [22]. Written informed consent was obtained from all participants.

## Results

### Socio-demographic and clinical characteristics

The socio-demographic characteristics of the study participants are presented in Table 4. One thousand, three hundred and ten (1,310) respondents comprising 767 (58.5%) males and 543 (41.5%) females participated in the study; the highest proportion, 757 (57.7%), were in the 20–39 years age group. Seven hundred and twenty-nine (55.6%) participants were married and 654 (49.9%) practised Islam. The Hausa ethnic group had the highest frequency (547; 41.8%) while 347 (26.5%) were unskilled labourers, and 767 (58.5%) had higher than secondary school education. A total of 864 (65.1%) participants presented to the testing sites with at least one COVID-19 related symptom.

**Table 4:** Sociodemographic characteristics of participants (N = 1310).

Characteristics	Frequency	Percentage
**Sex**		
Male	767	58.5
Female	543	41.5
**Age group (in years)**		
0–9	21	1.6
10–19	66	5.0
20–29	366	27.9
30–39	391	29.8
40–49	187	14.3
50–59	117	8.9
60–69	83	6.3
70–79	51	3.9
≥80	19	1.5
No response	9	0.7
**Marital status**		
Married	729	55.6
Single	516	39.4
Widow	22	1.7
Widower	9	0.7
Separated	3	0.2
No response	19	1.5
**Religion**		
Islam	654	49.9
Christianity	611	46.6
Traditional Religion	8	0.6
No response	37	2.8
**Ethnicity**		
Hausa	547	41.8
Yoruba	267	20.4
Igbo	121	9.2
Others^∞^	341	26.0
No response	34	2.6
**Occupation**		
Unskilled Labour	347	26.5
Professional/technical (Excluding health care workers)	241	18.4
Unemployed (Student)	187	14.3
Skilled Labour	188	14.4
Health care workers	173	13.2
Retired	91	6.9
No response	83	6.3
**Highest level of Education**		
No formal Education	151	11.5
Primary	82	6.3
Secondary	269	20.5
Higher Education	767	58.5
No response	41	3.1
**Clinical Status**		
Symptomatic	864	65.1
Asymptomatic	359	27.1
No response	104	7.8
**Consortium site** ‡		
OAU	457	34.9
UNIBEN	207	15.8
FMC Abeokuta	173	13.2
IHVN	135	10.3
U.I	126	9.6
JUTH	121	9.2
ZRC	91	6.9

^∞^-Others indicate about 104 ethnic tribes,

^‡^: OAU–Obafemi Awolowo University; UNIBEN–University of Benin; FMC Abeokuta–Federal Medical Centre, Abeokuta; IHVN—Institute of Human Virology, Nigeria; UI–University of Ibadan; JUTH–Jos University Teaching Hospital; ZRC- Zankli Research Centre, Bingham University Karu

The clinical symptoms reported by the study participants are presented in Table 5. The three most common symptoms reported were: fever (701, 53.5%), dry cough (659; 50.3%), and tiredness (458; 35.0%). Less commonly reported symptoms were sore throat (185; 14.1%), difficulty in breathing (114; 8.7%), and diarrhoea (67; 5.1%).

**Table 5:** Clinical symptoms of the study participants (N = 1310).

Characteristics	Frequency	Percentage
**Fever**		
No	549	41.9
Yes	701	53.5
No response	60	4.6
**Dry Cough**		
No	592	45.2
Yes	659	50.3
No response	59	4.5
**Difficulty in breathing**		
No	1138	86.9
Yes	114	8.7
No response	58	4.4
**Tiredness**		
No	789	60.2
Yes	458	35.0
No response	63	4.8
**Aches and pains**		
No	1052	80.3
Yes	193	14.7
No response	65	5.0
**Nasal Congestion**		
No	1005	76.7
Yes	243	18.5
No response	62	4.7
**Runny nose**		
No	933	71.2
Yes	313	23.9
No response	64	4.9
**Diarrhoea**		
No	1179	90.0
Yes	67	5.1
No response	64	4.9
**Sore throat**		
No	1062	81.1
Yes	185	14.1
No response	63	4.8

### Description of RDT diagnostic results

Table 6 summarises the diagnostic performance of six antigen-based RDT kits (Lifotronic, Lumira Dx, Mologic, MP SARS, Panbio, and SGTI-Flex) for COVID-19 diagnosis/screening compared to the gold-standard test (RT-PCR) in terms of area under ROC curve (AUROC) values, sensitivity, specificity, positive predictive value (PPV), and negative predictive value (NPV). Apart from Lumira Dx with an AUROC value of 0.79 (95% CI: 0.74–0.83), the other antigen-based kits had AUROC values that were less than 0.7.

**Table 6:** Diagnostic accuracy of antigen-based RDT kits for COVID-19 diagnosis/screening compared to PCR test for combined participants.

Antigen RDT Kit‡	AUROC (95% CI)	Sensitivity (95% CI)	Specificity (95% CI)	PPV (95% CI)	NPV (95% CI)
Lifotronic (n = 561)	0.68 (0.64–0.71)	37.4 (30.5–44.8)	97.9 (95.8–99.1)	89.7 (80.8–95.5)	75.8 (71.7–79.5)
Lumira Dx (n = 347)	**0.79** **(0.74–0.83)**	**61.4** **(52.4–69.9)**	95.9 (92.4–98.1)	89.7 (81.3–95.2)	**81.2** **(75.9–85.7)**
Mologic (n = 488)	0.63 (0.60–0.67)	28.8 (22.6–35.6)	97.9 (95.6–99.2)	90.5 (80.4–96.4)	66.8 (62.1–71.3)
MP SARS (n = 515)	0.63 (0.59–0.66)	26.8 (20.5–33.9)	**98.5** **(96.6–99.5)**	90.6 (79.3–96.9)	71.6 (67.3–75.7)
Panbio (n = 493)	0.64 (0.61–0.68)	29.9 (23.0–37.5)	**98.5** **(96.5–99.5)**	**90.7** **(79.7–96.9)**	73.8 (69.4–77.9)
SGTI-Flex (n = 337)	0.59 (0.55–0.64)	21.2 (13.1–31.4)	97.2 (94.4–98.9)	72.0 (50.6–87.9)	78.5 (73.5–83.0)

AUROC = Area under the receiver operating characteristics curve; PPV = Positive Predictive Value; NPV = Negative Predictive Value

^‡^Calculation of predictive scores requires complete observations for both RDT and PCR, hence the variations in sample size

In terms of diagnostic performance, Lumira Dx (61.4, 95% CI: 52.4–69.9) had the highest sensitivity while MP SARS and Panbio (98.5, 95% CI: 96.6–99.5) had the highest specificity. In terms of predictive value, Panbio (90.7, 95% CI: 79.7–96.9) and Lumira Dx (81.2, 95% CI: 75.9–85.7) recorded the highest PPV and NPV in diagnosing/screening COVID-19 respectively.

Sub-group analysis of the diagnostic accuracy of Ag RDTs on symptomatic patients (Table 7) revealed that Lumirax Dx had the highest AUROC (0.83, 95% CI: 0.78–0.89), sensitivity (71.8, 95% CI: 59.9–81.9), and NPV (87.7, 95% CI: 81.7–92.3) values. The specificity values for all Ag RDT kits validated were higher than 90% for symptomatic patients.

**Table 7:** Diagnostic accuracy of antigen-based RDT kits for COVID-19 diagnosis/screening compared to PCR test for symptomatic participants.

Antigen RDT Kit‡	AUROC (95% CI)	Sensitivity (95% CI)	Specificity (95% CI)	PPV (95% CI)	NPV (95% CI)
Lifotronic (n = 353)	0.72 (0.67–0.77)	46.8 (37.3–56.6)	**97.5** **(94.7–99.1)**	89.7 (78.8–96.1)	80.0 (75.0–84.4)
Lumira Dx (n = 222)	**0.83** **(0.78–0.89)**	**71.8** **(59.9–81.9)**	94.7 (89.8–97.7)	86.4 (75.0–94.0)	**87.7** **(81.7–92.3)**
Mologic (n = 278)	0.67 (0.63–0.72)	38.0 (29.3–47.3)	96.8 (92.7–99.0)	**90.2** **(78.6–96.7)**	67.0 (60.4–73.0)
MP SARS (n = 305)	0.67 (0.62–0.72)	36.9 (27.6–47.0)	**97.5** **(94.3–99.2)**	88.4 (74.9–96.1)	75.2 (69.5–80.3)
Panbio (n = 301)	0.68 (0.63–0.73)	38.1 (28.5–48.6)	**97.5** **(94.4–99.2)**	88.1 (74.4–96.0)	76.8 (71.2–81.8)
SGTI-Flex (n = 209)	0.61 (0.55–0.67)	25.0 (14.1–38.4)	97.4 (93.4–99.3)	77.8 (52.4–93.6)	78.0 (71.5–83.7)

AUROC = Area under the receiver operating characteristics curve; PPV = Positive Predictive Value; NPV = Negative Predictive Value

^‡^Calculation of predictive scores requires complete observations for both RDT and PCR, hence the variations in sample size

Analysis of the asymptomatic individuals showed that Lumirax Dx had the highest AUROC value (0.77, 95% CI: 0.68–0.86) and sensitivity (56.3, 95% CI: 37.7–73.6) values. MP SARS and Panbio Ag RDTs recorded 100% specificity and PPVs; while SGTI-Flex recorded the highest NPV (84.8, 95% CI: 76.4–91.0)) (Table 8).

**Table 8:** Diagnostic accuracy of antigen-based RDT kits for COVID-19 diagnosis/screening compared to PCR test for asymptomatic participants.

Antigen RDT Kit‡	AUROC (95% CI)	Sensitivity (95% CI)	Specificity (95% CI)	PPV (95% CI)	NPV (95% CI)
Lifotronic (n = 169)	0.61 (0.55–0.67)	22.9 (12.0–37.3)	99.2 (95.5–100.0)	91.7 (61.5–99.8)	76.4 (69.0–82.8)
Lumira Dx (n = 93)	**0.77** **(0.68–0.86)**	**56.3** **(37.7–73.6)**	98.4 (91.2–100.0)	94.7 (74.0–99.9)	81.1 (70.3–89.3)
Mologic (n = 169)	0.58 (0.53–0.63)	16.7 (7.5–30.2)	99.2 (95.5–100.0)	88.9 (51.8–99.7)	75.0 (67.6–81.5)
MP SARS (n = 172)	0.52 (0.49–0.55)	4.3 (0.5–14.8)	**100.0** **(97.1–100.0)**	**100.0** **(15.8–100.0)**	74.1 (66.9–80.5)
Panbio (n = 157)	0.59 (0.53–0.65)	17.5 (7.3–32.8)	**100.0** **(96.9–100.0)**	**100.0** **(59.0–100.0)**	78.0 (70.5–84.3)
SGTI-Flex (n = 108)	0.52 (0.46–0.58)	5.9 (0.1–28.7)	97.8 (92.3–99.7)	33.3 (0.8–90.6)	**84.8** **(76.4–91.0)**

AUROC = Area under the receiver operating characteristics curve; PPV = Positive Predictive Value; NPV = Negative Predictive Value

^‡^Calculation of predictive scores requires complete observations for both RDT and PCR, hence the variations in sample size

Table 9 summarises the diagnostic performance of the IgM component of four antibody-based RDT kits (Genrui, SD Biosensor, Sugentech, and Rightsign) for COVID-19 diagnosis/screening compared to the gold-standard test (PCR). Overall, all the IgM components of the kits had poor discriminative capacity for COVID-19 diagnosis, with AUROC values ranging from 0.51–0.59. Notably, SD Biosensor had the highest AUROC of 0.59. The sensitivity of the IgM detection kits was generally low, with Sugentech having the highest value of 30.0% (95%: CI 22.3–38.7). In contrast, the specificity of the kits was relatively high, with the highest value of 95.0% (95% CI: 92.1–97.0) observed in the Genrui IgM kit, which also had the highest NP (75.5%) values, albeit both values are relatively low.

**Table 9:** Diagnostic accuracy of antibody-based (IgM) RDT kits for COVID-19 diagnosis/screening compared to PCR test for combined participants.

Antibody RDT Kit	AUROC (95% CI)	Sensitivity (95% CI)	Specificity (95% CI)	PPV (95% CI)	NPV (95% CI)
Genrui (n = 454)	0.53 (0.50–0.56)	10.3 (5.5–17.4)	**95.0** **(92.1–97.0)**	41.4 (23.5–61.1)	**75.5** **(71.2–79.5)**
Sugentech (n = 367)	0.51 (0.46–0.56)	**30.0** **(22.3–38.7)**	72.6 (66.4–78.2)	37.5 (28.2–47.5)	65.4 (59.3–71.1)
Rightsign (n = 394)	0.53 (0.50–0.57)	15.5 (9.3–23.6)	91.2 (87.3–94.2)	40.5 (25.6–56.7)	73.6 (68.6–78.1)
SD Biosensor (n = 316)	**0.59** **(0.54–0.63)**	25.0 (17.2–34.3)	92.3 (87.8–95.5)	**62.8** **(46.7–77.0)**	70.3 (64.5–75.7)

AUROC = Area under the receiver operating characteristics curve; PPV = Positive Predictive Value; NPV = Negative Predictive Value

Concerning sub-group analysis, SD Biosensor IgM RDT had the highest AUROC (0.60, 95% CI: 0.55–0.66); Sugentech had the highest sensitivity (31.6, 95% CI: 22.6–41.8), while Genrui had the highest specificity (95.2, 95% CI: 91.0–97.8). The highest PP (69.4, 95% CI: 51.9–83.7) and NP (70.0, 95% CI: 63.9–75.5)) values for symptomatic participants were recorded by SD Biosensor and Genrui respectively (Table 10).

**Table 10:** Diagnostic accuracy of antibody-based (IgM) RDT kits for COVID-19 diagnosis/screening compared to PCR test for symptomatic participants.

Antibody RDT Kit	AUROC (95% CI)	Sensitivity (95% CI)	Specificity (95% CI)	PPV (95% CI)	NPV (95% CI)
Genrui (n = 272)	0.53 (0.50–0.57)	11.6 (5.7–20.3)	**95.2** **(91.0–97.8)**	52.6 (28.9–75.6)	**70.0** **(63.9–75.5)**
Sugentech (n = 218)	0.52 (0.46–0.58)	**31.6** **(22.6–41.8)**	71.7 (62.7–79.5)	47.7 (35.1–60.5)	56.2 (48.0–64.2)
Rightsign (n = 232)	0.55 (0.50–0.60)	19.3 (11.4–29.4)	91.3 (85.5–95.3)	55.2 (35.7–73.6)	67.0 (60.1–73.4)
SD Biosensor (n = 203)	**0.60** **(0.55–0.66)**	29.8 (20.3–40.7)	90.8 (84.1–95.3)	**69.4** **(51.9–83.7)**	64.7 (56.9–71.9)

AUROC = Area under the receiver operating characteristics curve; PPV = Positive Predictive Value; NPV = Negative Predictive Value

The AUROC values for all IgM RDTs for asymptomatic participants were also low; with Genrui and SD Biosensor giving the highest value of 0.49. The sensitivity values for this sub-group were much lower than for the symptomatic group–the highest value recorded was 23.3% for Sugentech RDT. Compared to the symptomatic group, the specificity for the asymptomatic participants was higher–with Genrui and SD Biosensor RDT giving the highest value of 94.4%. The PP values were low—Sugentech (18.4%); while the highest NPV was (84.0%) recorded from the Genrui RDT (Table 11).

**Table 11:** Diagnostic accuracy of antibody-based (IgM) RDT kits for COVID-19 diagnosis/screening compared to PCR test for asymptomatic participants.

Antibody RDT Kit	AUROC (95% CI)	Sensitivity (95% CI)	Specificity (95% CI)	PPV (95% CI)	NPV (95% CI)
Genrui (n = 171)	**0.49** **(0.45–0.53)**	3.7 (0.1–19.0)	**94.4** **(89.3–97.6)**	11.1 (0.3–48.2)	**84.0** **(77.4–89.2)**
Sugentech (n = 144)	0.48 (0.39–0.57)	**23.3** **(9.9–42.3)**	72.8 (63.7–80.7)	**18.4** **(7.7–34.3)**	78.3 (69.2–85.7)
Rightsign (n = 152)	0.48 (0.43–0.52)	4.0 (0.1–20.4)	91.3 (85.0–95.6)	8.3 (0.2–38.5)	82.9 (75.6–88.7)
SD Biosensor (n = 112)	**0.49** **(0.44–0.54)**	4.3 (0.1–21.9)	**94.4** **(87.4–98.2)**	16.7 (0.4–64.1)	79.2 (70.3–86.5)

AUROC = Area under the receiver operating characteristics curve; PPV = Positive Predictive Value; NPV = Negative Predictive Value

Table 12 summarises the diagnostic performance of the IgG component of four antibody-based RDT kits (Genrui, SD Biosensor, Sugentech, and Rightsign) for COVID-19 diagnosis compared to the gold-standard test (PCR). Similar to the performance of the IgM detection, all the IgG-based kits showed a poor predictive capacity for COVID-19 diagnosis with AUROC values ranging from 0.51 to 0.57. Specifically, SD Biosensor’s IgG component had the highest AUROC at 0.57 (95%: 0.51–0.62). In terms of performance, SD Biosensor (44.4, 95% CI: 34.9–54.3) and Sugentech (81.1, 95% CI: 75.5–85.9) showed the highest sensitivity and specificity respectively while SD Biosensor (42.5, 95% CI: 33.2–52.1) and Genrui (77.4, 95% CI: 72.7–81.7) had the highest PPV and NPV respectively.

**Table 12:** Diagnostic accuracy of antibody-based (IgG) RDT kits for COVID-19 diagnosis/screening compared to PCR test for combined participants.

Antibody RDT Kit	AUROC (95% CI)	Sensitivity (95% CI)	Specificity (95% CI)	PPV (95% CI)	NPV (95% CI)
Genrui (n = 453)	0.56 (0.51–0.61)	31.9 (23.6–41.2)	80.4 (75.8–84.5)	35.9 (26.7–46.0)	**77.4** **(72.7–81.7)**
Sugentech (n = 368)	0.51 (0.47–0.55)	20.8 (14.2–28.8)	**81.1** **(75.5–85.9)**	37.5 (26.4–49.7)	65.2 (59.5–70.6)
Rightsign (n = 394)	0.51 (0.46–0.56)	37.3 (28.2–47.0)	64.4 (58.6–70.0)	28.9 (21.6–37.1)	72.6 (66.7–78.0)
SD Biosensor (n = 317)	**0.57** **(0.51–0.62)**	**44.4** **(34.9–54.3)**	68.9 (62.1–75.1)	**42.5** **(33.2–52.1)**	70.6 (63.8–76.7)

AUROC = Area under the receiver operating characteristics curve; PPV = Positive Predictive Value; NPV = Negative Predictive Value

The separate analysis of symptomatic (0.56–0.61) and asymptomatic (0.42–0.54) participants revealed low AUROC values. Symptomatic participants (25.5–42.2) showed relatively higher sensitivity values than asymptomatic (6.7–47.8) ones. The specificity values for asymptomatic (59.6–83.5) and symptomatic (69.1–83.2) participants were similar. The PPV of the symptomatic (43.2–55.4) and asymptomatic (9.5–23.4) sub-categories were also relatively low. NPV for symptomatic participants ranged from 56.5–74.4; while that for asymptomatic subjects ranged from 77.4–82.8 (Tables 13 and 14).

**Table 13:** Diagnostic accuracy of antibody-based (IgG) RDT kits for COVID-19 diagnosis/screening compared to PCR test for symptomatic participants.

Antibody RDT Kit	AUROC (95% CI)	Sensitivity (95% CI)	Specificity (95% CI)	PPV (95% CI)	NPV (95% CI)
Genrui (n = 271)	**0.61** **(0.55–0.67)**	38.4 (28.1–49.5)	**83.2** **(77.1–88.3)**	51.6 (38.7–64.2)	**74.4** **(67.9–80.2)**
Sugentech (n = 218)	0.52 (0.47–0.58)	25.5 (17.2–35.3)	79.2 (70.8–86.0)	50.0 (35.5–64.5)	56.5 (48.7–64.2)
Rightsign (n = 232)	0.56 (0.49–0.62)	42.2 (31.4–53.5)	69.1 (61.0–76.4)	43.2 (32.2–54.7)	68.2 (60.1–75.5)
SD Biosensor (n = 203)	0.59 (0.53–0.66)	**42.9** **(32.1–54.1)**	75.6 (66.9–83.0)	**55.4** **(42.5–67.7)**	65.2 (56.6–73.1)

AUROC = Area under the receiver operating characteristics curve; PPV = Positive Predictive Value; NPV = Negative Predictive Value

**Table 14:** Diagnostic accuracy of antibody-based (IgG) RDT kits for COVID-19 diagnosis/screening compared to PCR test for asymptomatic participants.

Antibody RDT Kit	AUROC (95% CI)	Sensitivity (95% CI)	Specificity (95% CI)	PPV (95% CI)	NPV (95% CI)
Genrui (n = 171)	0.46 (0.38–0.54)	14.8 (4.2–33.7)	77.1 (69.3–83.7)	10.8 (3.0–25.4)	**82.8** **(75.4–88.8)**
Sugentech (n = 145)	0.45 (0.39–0.51)	6.7 (0.8–22.1)	**83.5** **(75.4–89.7)**	9.5 (1.2–30.4)	77.4 (69.0–84.4)
Rightsign (n = 152)	0.42 (0.32–0.51)	24.0 (9.4–45.1)	59.1 (50.0–67.7)	10.3 (3.9–21.2)	79.8 (70.2–87.4)
SD Biosensor (n = 112)	**0.54** **(0.42–0.65)**	**47.8** **(26.8–69.4)**	59.6 (48.6–69.8)	**23.4** **(12.3–38.0)**	81.5 (70.0–90.1)

AUROC = Area under the receiver operating characteristics curve; PPV = Positive Predictive Value; NPV = Negative Predictive Value

A combined forest plot of the sensitivities and specificities of RDTs evaluated in this study is presented in the S1 Table.

## Discussion

To the best of our knowledge, this is the largest multi-centre study that evaluated a significant number of commercially available antigen and antibody-based rapid diagnostic test (RDTs) kits including some that have not been previously evaluated in Africa. Despite widespread deployment, there is a dearth of information about the diagnostic accuracy of RDTs for COVID-19 diagnosis in Africa. Compared to the reference test (RT-PCR), Ag-RDTs evaluated in this study appeared to perform better than antibody-based tests in the diagnosis of COVID-19. Although all Ag-RDTs showed good specificity with most exceeding 97%, their sensitivities were far below the benchmark of at least 80% set by WHO [9] This observation implies that many positive cases will be missed thus leading to further transmission and spread of the virus. The individual and collective sensitivity of Ag-RDTs found in this study were below the range of values documented in some studies from other African countries [10–14, 23]. This may be attributed to differences in the period of the pandemic when the study was done as well the circulating variants at the time of testing and evaluation. Most of the other African studies were conducted earlier in the pandemic (2020), unlike our study which spanned the last quarter of 2021 and early 2022. Thus, the differences in sensitivity may be related to the difference in the circulating variants at the time of the study compared to the variant from which the reagents were produced. Antigen detection tests are generally less sensitive and less likely to pick up very early infections compared to molecular tests. In the USA, the FDA identified certain EUA-authorized antigen tests whose performance may be impacted by mutations in the SARS-CoV-2 and some of these tests were modified accordingly [24].

Several studies have described reduced sensitivity of Ag-RDTs to the alpha, delta, and omicron variants even after adjusting for other conditions affecting sensitivity such as Ct values and the presence of clinical symptoms [25–28]. It is worthy to note that the predominant circulating variants in this study were Delta prior to December 2021 and Omicron after that. On the other hand, more recent studies have implicated immunity from vaccination and previous infection as opposed to virus variants as a reason for the decreasing sensitivity of Ag-RDT [29]. Thus, variation in the seroprevalence levels in the different study countries may account for the marked differences in sensitivity observed. The relative proportion of asymptomatic persons, persons with extended time post-symptom onset, and low viral loads may also have contributed to the combined low sensitivity values obtained in this study; this is reflected in the higher sensitivity observed in symptomatic versus asymptomatic individuals after sub-group analysis.

In general, diagnostic accuracy data from Africa show lower sensitivity than reports from Europe and America [30]. Khandker *et al*. [30] have speculated that the performance of these tests may be affected by freeze-thaw cycles (for reagents requiring cold chain) during transportation to Africa and Asia from foreign manufacturers. They proffered local manufacturing as the solution to the possible deterioration associated with transportation. It is also important to note that the RDT kits detect the antigen and not the nuclear material, which is the target for the PCR test (Gold standard). The nuclear material (viral RNA) may be detected even when a viable virus is not present, unlike the situation with a viral antigen which is an indication of the presence of a viable virus. Also, viral antigens are subject to the immune response of the host. These reasons may explain the differences in the detection rate by RDTs and PCR and thus the lower sensitivity of the RDTs.

Four antibody-based RDTs were also evaluated for their suitability for COVID-19 diagnosis in this study. Their overall poor performance is not surprising as antibody tests measure the body’s immune response to the virus rather than the presence of the virus itself which is the target of the gold standard PCR. In the early months of the pandemic, and against a background of absent immunity to a new disease, antibody tests were widely evaluated as diagnostic tools for COVID-19 [31]. However, their usefulness was limited by delayed and poor predictability of the timing of antibody appearance in the serum. This limitation has been further compounded by the build-up of immunity from past infections and vaccination as the pandemic progressed. In our study, nevertheless, the specificity of the IgM component of antibody-based RDTs was generally higher than that of IgG, implying that this aspect of the tests may have some utility in detecting recent infections and may find use in orthogonal testing algorithms. A study carried out by WHO collaborating centre in Belgium reported cross-reactivity of SARS-CoV-2 antibodies of 20.3%, 18.5% and 7.5% with malaria, schistosomiasis, and dengue respectively; however, this cross-reactivity was not associated with detecting the SARS-CoV-2 antigen [32]. While we do not have specific data for each of the study’s locations, malaria (20 70%) [33], schistosomiasis (44.8% to 71.5) [34], and dengue (30.8%) [35] are prevalent in Nigeria. However, our study did not investigate their possible effects on the performance of the various assays.

### Limitations

A major strength of this study is its multi-centred nature which makes the data representative of the entire country. The study, however, has a few limitations, which include: (1) Due to the need to minimize contact with suspected SARS-CoV-2 exposed individuals, some demographic data and clinical symptoms were collected using self-administered questionnaires with the resultant ‘non-response’ (2) a comprehensive head-to-head comparison of the Ag-RDTs was not feasible because some enrolees did not consent to the collection of multiple swabs; (3) comparability of the results may be limited by the non-uniformity in SARS-CoV-2 assays employed for molecular diagnosis at the various sites; and (4) RT-PCR tests, despite being the gold standard, is known to detect a non-viable viral nucleic acid. This may result in an erroneous perception of low performance in the RDTs.

### Recommendations

Limitations notwithstanding, findings from our study have implications for the use of rapid diagnostic tests for COVID-19 diagnosis in Nigeria. Antibody-based RDTs may have limited use in detecting acute disease but they may be useful in the surveillance of recent and past COVID-19 infections, this is supported by the higher sensitivity values obtained for symptomatic individuals in this study. Ag-RDTs may be used to quickly detect COVID-19 at reduced cost in clinical settings but negative results must be interpreted cautiously and supplemented with additional PCR testing where clinical suspicion is high. The use of these tests as screening tools at international ports of entry, campgrounds, and mass gatherings is not recommended, especially during periods of high transmission, as many positive persons would be missed resulting in untoward transmission.

In summary, the RDTs evaluated are not sensitive enough to replace RT-PCR for the diagnosis of COVID-19. However, the high specificity of Ag-RDTs makes them useful supplements capable of reducing the need for molecular testing in confirming COVID-19-positive samples in Nigeria.

### Further research

More studies are required to evaluate the effect of temperature, other prevailing infections such as malaria, virus strains, and population immunity on the performance of these tests across diverse settings. The need for the local development of SAR-CoV-2 RDTs with improved sensitivity and specificity is also recommended.

## Supporting information

S1 File (DOCX)

S1 Table (DOCX)

S1 Checklist STARD checklist. (DOCX)

S2 Checklist STROBE checklist. (DOCX)
