# Supplementary material for: Performance evaluation of SARS-CoV-2 rapid diagnostic tests in Nigeria: A cross-sectional study
Source: PLOS Glob Public Health. 2024 Jul 15;4(7):e0003371. doi: 10.1371/journal.pgph.0003371 (PMC11249252; doi:10.1371/journal.pgph.0003371)
Supplement: S1 File — (DOCX) [file pgph.0003371.s001.docx]

**Members of COVID-19 RDT Validation in Nigeria (CORVAN) Study Group**

| **S/N** | **NAME** | **AFFILIATION** |
| --- | --- | --- |
| 1 | Prof. Akhere A. Omonkhua | University of Benin |
| 2 | Dr Iriagbonse Iyabo Osaigbovo | University of Benin Teaching Hospital |
| 3 | Dr. Kelly Elimian | University of Benin, Nigeria COVID-19 Research Coalition |
| 4 | Dr Cornelius Ohonsi | Nigeria Centre for Disease Control & Prevention, Nigeria COVID-19 Research Coalition |
| 5 | Dr Temitope Ojo | Obafemi Awolowo University, Ile-ife |
| 6 | Dr. Nnadi Emmanuel | Jos University Teaching Hospital |
| 7 | Prof. John Aguiyi. PhD | University of Jos, Plateau State |
| 8 | Dr. Luka Pam D. | National Veterinary Research Institute (NVRI) Vom |
| 9 | Dr. Shwe David | National Veterinary Research Institute (NVRI) Vom |
| 10 | Dr. Omale Simon | National Veterinary Research Institute (NVRI) Vom |
| 11 | Dr. Afolaranmi Tolulupe | National Veterinary Research Institute (NVRI) Vom |
| 12 | Dr. Gomerep Simji: | National Veterinary Research Institute (NVRI) Vom |
| 13 | Dr. Izang, Joy Abi | West African Center for Emerging Infectious Disease (WAC-EID) |
| 14 | Dr. Emmanuel Ameh | National Veterinary Research Institute (NVRI) Vom |
| 15 | Mr. Moses Longji Dashal | National Veterinary Research Institute (NVRI) Vom |
| 16 | Mr. Elisha Abare | National Veterinary Research Institute (NVRI) Vom |
| 17 | Sanda Antun | National Veterinary Research Institute (NVRI) Vom |
| 18 | Bitrus Longkem | National Veterinary Research Institute (NVRI) Vom |
| 19 | Joseph Musa | National Veterinary Research Institute (NVRI) Vom |
| 20 | Dr. Ismaila Shittu | National Veterinary Research Institute (NVRI) Vom |
| 21 | Dr. Bitrus Inuwa | National Veterinary Research Institute (NVRI) Vom |
| 22 | Dr. Mark Samson | National Veterinary Research Institute (NVRI) Vom |
| 23 | Miss Helen G. Luka | National Veterinary Research Institute (NVRI) Vom |
| 24 | Dr. Yusuf Bara Jibrin | Abubakar Tafawa Balewa University Teaching Hospital (ATBUTH) |
| 25 | Dr. Ibrahim Mahmood Maigari | Abubakar Tafawa Balewa University Teaching Hospital (ATBUTH) |
| 26 | Dr. Sulaiman Alhaji | Abubakar Tafawa Balewa University Teaching Hospital (ATBUTH) |
| 27 | Dr. Hamisu Abdulrasheed | Abubakar Tafawa Balewa University Teaching Hospital (ATBUTH) |
| 28 | Dr. Hafiza sulaiman | Abubakar Tafawa Balewa University Teaching Hospital (ATBUTH) |
| 29 | Aisha Muhammad Malami | Abubakar Tafawa Balewa University Teaching Hospital (ATBUTH) |
| 30 | Massa Edna Makinba | Abubakar Tafawa Balewa University Teaching Hospital (ATBUTH) |
| 31 | Mr. Abdullahi Abubakar | Institute of Human Virology, Nigeria |
| 32 | Mr. Anthony Ahumide | Nigeria Centre for Disease Control |
| 33 | Dr. James Onyemata | Institute of Human Virology, Nigeria |
| 34 | Mrs.Teclaire Ngondomb | Institute of Human Virology, Nigeria |
| 35 | Mrs. Stella Ijioma | Institute of Human Virology, Nigeria |
| 36 | Mr. Remi Are | Institute of Human Virology, Nigeria |
| 37 | Onisile Oluwaseun | Institute of Human Virology, Nigeria |
| 38 | Mrs. Charity Sanni | Institute of Human Virology, Nigeria |
| 39 | Mr. Femi Omoniji | Institute of Human Virology, Nigeria |
| 40 | Mr. George Odonye | Institute of Human Virology, Nigeria |
| 41 | Ms. Sophia Chiamaka Ukwenga | Institute of Human Virology, Nigeria |
| 42 | Stephen Oricha | University of Abuja Teaching Hospital |
| 43 | Chinelo Udemezue | University of Abuja Teaching Hospital |
| 44 | Dr Omosivie Maduka | University of Port Harcourt teaching Hospital |
| 45 | Abimbola Awopeju | University of Port Harcourt teaching Hospital |
| 46 | Emuh Faith | University of Port Harcourt teaching Hospital |
| 47 | Franklin Osinanna | University of Port Harcourt teaching Hospital |
| 48 | Lily chioma | University of Port Harcourt teaching Hospital |
| 49 | Ifeoma Rapheal | University of Port Harcourt teaching Hospital |
| 50 | Chioma Ephraim | University of Port Harcourt teaching Hospital |
| 51 | Kushim Jonathan Agwom | Zankli Research Center, Bingham University, Karu |
| 52 | Kasimu Aliyu | Zankli Research Center, Bingham University, Karu |
| 53 | Timan Eliya | Zankli Research Center, Bingham University, Karu |
| 54 | Rose B. Abiola | Zankli Research Center, Bingham University, Karu |
| 55 | Precious Ishaku | Zankli Research Center, Bingham University, Karu |
| 56 | Godiya Moses | Zankli Research Center, Bingham University, Karu |
| 57 | John Abraham | Zankli Research Center, Bingham University, Karu |
| 58 | Dr Tunde Olusola | University of Ibadan |
| 59 | Dr Adedayo Faneye | University of Ibadan |
| 60 | Mr Olawale Opayele | University of Ibadan |
| 61 | Mr Femi Olarogba | University of Ibadan |
| 62 | Mrs Oluwaposi Oke | University of Ibadan |
| 63 | Mr Oluwaseun Ala | University of Ibadan |
| 64 | Mrs Dupe Adewuyi | University of Ibadan |
| 67 | Mrs Bukola Ajagbe | University of Ibadan |
| 66 | Mrs Oluwaseun Olaniyan | University of Ibadan |
| 67 | Mr Mohammed Mukhtar | University of Ibadan |
| 68 | Mr Joel Aluko | University of Ibadan |
| 69 | Mr Clement Adedigba | University of Ibadan |
| 70 | Bisi Makinde | University of Ibadan |
| 71 | Dr Elisabeth Odeh | Federal University Teaching Hospital Abakiliki Ebonyi State |
| 72 | Dr Okoro Sylvanus Ihere | Federal University Teaching Hospital Abakiliki Ebonyi State |
| 73 | Onyia Venatius Onyekachi | Federal University Teaching Hospital Abakiliki Ebonyi State |
| 74 | Dr. Loretta Ntoimo | University of Benin |
| 75 | Dr. Nosakhare Enaruna | University of Benin |
| 76 | Dr. Kenneth Maduako | University of Benin |
| 77 | Vivian Onoh | University of Benin |
| 78 | Mr. Lucky Ebaluegbeifoh | University of Benin |
| 79 | Mr Isaac Igbarumah | University Of Benin Teaching Hospital |
| 80 | Dr Emmanuel Oduware | University Of Benin Teaching Hospital |
| 81 | Dr Benson Okwara | University Of Benin Teaching Hospital |
| 82 | Dr Samuel Adebudo | University Of Benin Teaching Hospital |
| 83 | Dr Chika Enebeli | University Of Benin Teaching Hospital |
| 84 | Mr Joseph Egbe | University Of Benin Teaching Hospital |
| 85 | Mr John Oghaekor | University Of Benin Teaching Hospital |
| 86 | Prof Christian Happi | African Centre of Excellence for Genomics of Infectious Diseases (ACEGID) |
| 87 | Prof Onikepe Folarin | African Centre of Excellence for Genomics of Infectious Diseases (ACEGID) |
| 88 | Dr Johnson Okolie | African Centre of Excellence for Genomics of Infectious Diseases (ACEGID) |
| 89 | Dr Kazeem Akano | African Centre of Excellence for Genomics of Infectious Diseases (ACEGID) |
| 90 | Mrs Philomena Eromon | African Centre of Excellence for Genomics of Infectious Diseases (ACEGID) |
| 91 | Ms Jessica Uwanibe | African Centre of Excellence for Genomics of Infectious Diseases (ACEGID) |
| 91 | Ms Motunrayo Bejide | African Centre of Excellence for Genomics of Infectious Diseases (ACEGID) |
| 93 | Ms Grace Chukwu | African Centre of Excellence for Genomics of Infectious Diseases (ACEGID) |
| 94 | Mr Ahmed Mohammed | African Centre of Excellence for Genomics of Infectious Diseases (ACEGID) |
| 95 | Mr Oludayo Ope-Ewe | African Centre of Excellence for Genomics of Infectious Diseases (ACEGID) |
| 96 | Lilian Nwozor | Nigeria COVID-19 Research Consortium |
| 97 | Dabri Ohanu | Nigeria Centre for Disease Control, Nigeria COVID-19 Research Consortium |
| 98 | Gloria Nwiyi Ogochukwu | Nigeria Centre for Disease Control, Nigeria COVID-19 Research Consortium |
| 99 | Prof. Ibrahim Kida | University of Maiduguri teaching Hospital, Maiduguri |
| 100 | James Mshelia | University of Maiduguri teaching Hospital, Maiduguri |
| 101 | Nuhu Philibus Asarya | University of Maiduguri teaching Hospital, Maiduguri |
| 102 | Sufyaan Mamman Shetimma | University of Maiduguri teaching Hospital, Maiduguri |
| 103 | Ibrhim Gutti | University of Maiduguri teaching Hospital, Maiduguri |
| 104 | Dr. Magaji A. Mahmoud | Rasheed Gbadamosi Specialist Hospital, Jigawa |
| 105 | Mr Nuraddeen Muhammad | Rasheed Gbadamosi Specialist Hospital, Jigawa |
| 106 | Mr. Abdulkadir Garba | Rasheed Gbadamosi Specialist Hospital, Jigawa |
| 107 | Mr. Mahmud Ubale | Rasheed Gbadamosi Specialist Hospital, Jigawa |
| 108 | Mr. Murtala Sani Musa | Rasheed Gbadamosi Specialist Hospital, Jigawa |
| 109 | Dr Paul Akinduti | Department of Microbiology, Covenant University, Ota, Ogun State, Nigeria |
| 110 | Dr. Victory Fabian Edem | Department of Immunology, University of Ibadan, Ibadan, Nigeria |
| 111 | Dr. Onyeaghala Augustine | Dept. of Chemical Pathology, University College Hospital, Ibadan Nigeria |
| 112 | Dr. (Mrs) Kemi Ladipo | Babcock University, Ilishan Remo |
| 113 | Dr. Abdul Rahman El-Fulatty | Department of Medical Laboratory Science, Ahmadu Bello University, Zaria |
| 114 | Mohammed Tahir I. | Department of Medical Laboratory Science, Ahmadu Bello University, Zaria |
| 115 | Emmanuel Adebayo | Federal Medical Centre, Abeokuta |
| 116 | Isiaq Adeniyi | Federal Medical Centre, Abeokuta |
| 117 | Oyedeji Mobolaji (Miss) | Federal Medical Centre, Abeokuta |
| 118 | Aishat Alarape | Federal Medical Centre, Abeokuta |
| 119 | Olojede Adams | Ogun State Molecular Lab |
| 120 | Oladeji Sofoluke | Ogun State Molecular Lab |
| 121 | Wasiu Lawal | Ogun State Molecular Lab |
| 122 | Adeola Olawole | Ogun State Molecular Lab |
| 123 | Kemiki Olalekan A. | Babcock University, Ilishan Remo |
| 124 | Adeniyi John O. | Babcock University, Ilishan Remo |
| 125 | Omisakin Akinsola | Babcock University, Ilishan Remo |
| 126 | Dr. Okafor Patrick A. | School of Medical Laboratory Sciences, A. B. U Teaching Hospital Zaria. |
| 127 | Idris Muhammad | Hajiya Gambo Sawaba General Hospital Kofan Gayan Zaria |
| 128 | Dr. Michael Olugbile | The World Bank |
| 129 | Dr. Akinwande Kazeem S. | Federal Medical Centre, Abeokuta |
| 130 | Dr. John S. Bimba | Zankli Research Center, Bingham University, Karu |
| 131 | Dr. Shehu Nathan Y. | Jos University Teaching Hospital |
| 132 | Dr. Okpokoro Evaezi | Institute of Human Virology, Nigeria |
| 133 | Professor David Olaleye | University of Ibadan |
| 134 | Professor Georgina Odaibo | University of Ibadan |
| 135 | Prof. Adedeji Onayade | Obafemi Awolowo University, Ile-ife |
| 136 | Dr Chinwe Lucia Ochu | Nigeria Centre for Disease Control & Prevention, Nigeria COVID-19 Research Consortium |
| 137 | Prof Ehimario Igumbor | Centre for Infectious Disease Research, Nigerian Institute of Medical Research  Lagos, Nigeria, Nigeria COVID-19 Research Coalition |
| 138 | Prof. Friday Okonofua | University of Benin Teaching Hospital |
| 139 | Prof. Darlington Ewaen Obaseki | University of Benin Teaching Hospital |
| 140 | Dr Ifedayo Adetifa | Nigeria Centre for Disease Control & Prevention , Nigeria COVID-19 Research Coalition |
| 141 | Professor Babatunde L. Salako | Nigerian Institute of Medical Research, Nigeria COVID-19 Research Coalition |
| 142 | Dr. Chikwe Ihekweazu | World Health Organization, Nigeria COVID-19 Research Coalition |
| 143 | Dr. Mustapha Ayo Popoola, | Tertiary Education Trust Fund, Nigeria COVID-19 Research Coalition |
| 144 | Professor Suleiman Bogoro | Tertiary Education Trust Fund, Nigeria COVID-19 Research Coalition |
