# Supplementary material for: Performance evaluation of SARS-CoV-2 rapid diagnostic tests in Nigeria: A cross-sectional study
Source: PLOS Glob Public Health. 2024 Jul 15;4(7):e0003371. doi: 10.1371/journal.pgph.0003371 (PMC11249252; doi:10.1371/journal.pgph.0003371)
Supplement: S1 Table — (DOCX) [file pgph.0003371.s002.docx]

**Supplementary Table 1 - Composite forest plots of the sensitivities and specificities of antigen and antibody rapid diagnostic tests for all participants**


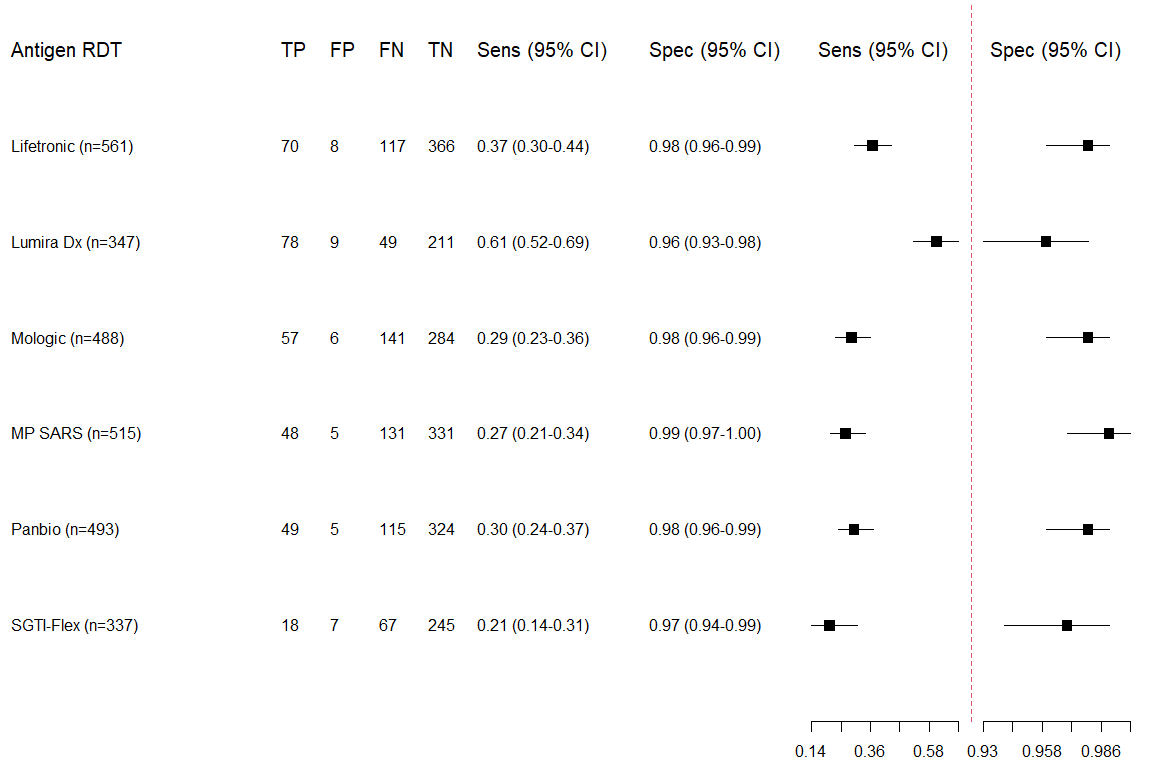


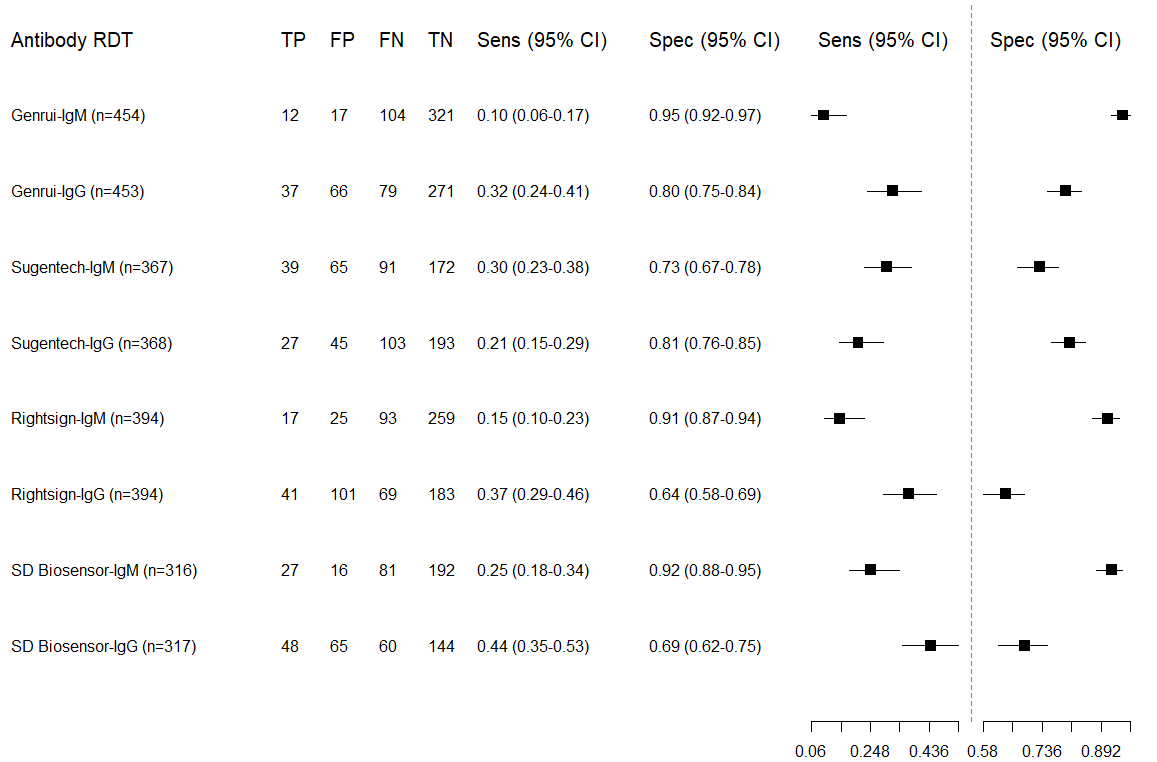


**Symptomatic Participants**


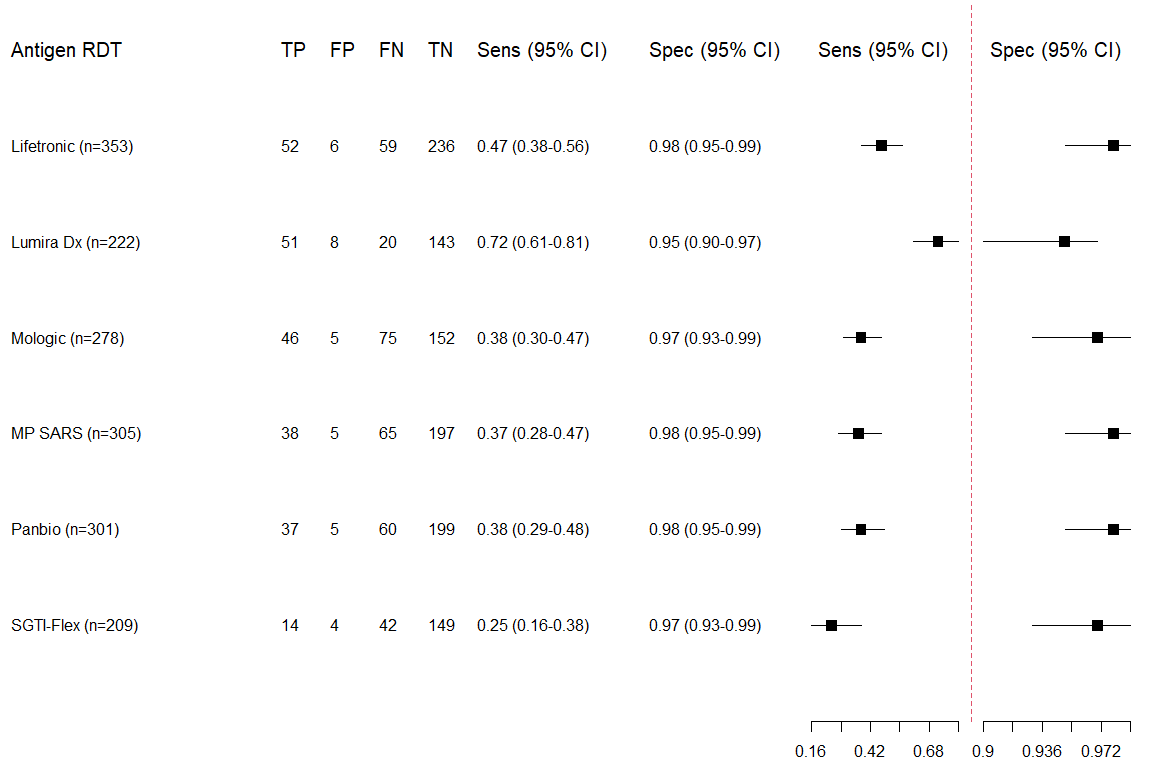


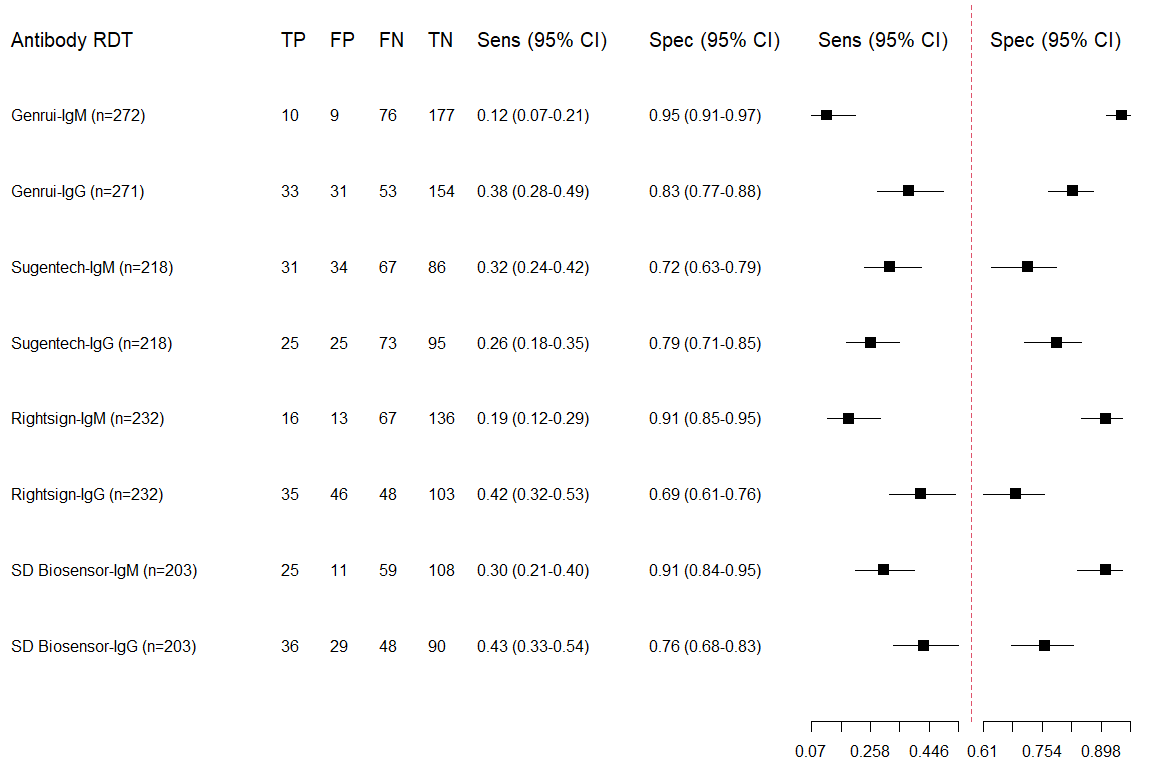


**Asymptomatic Participants**


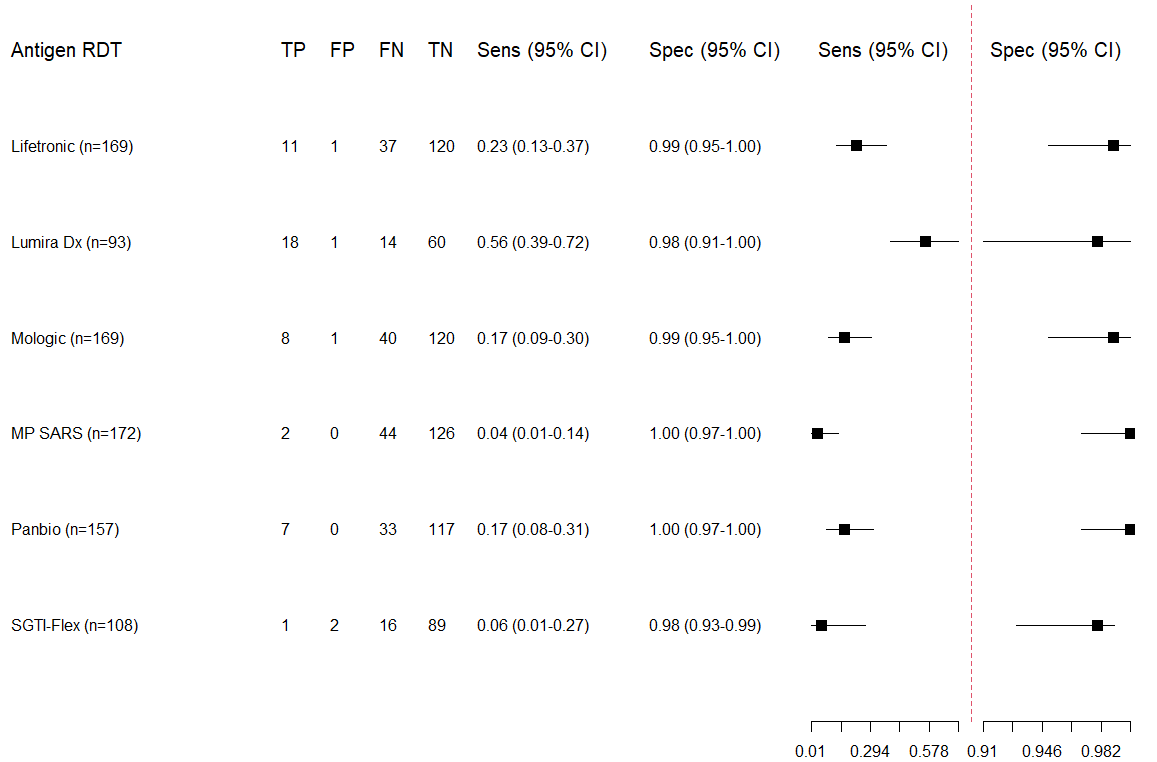


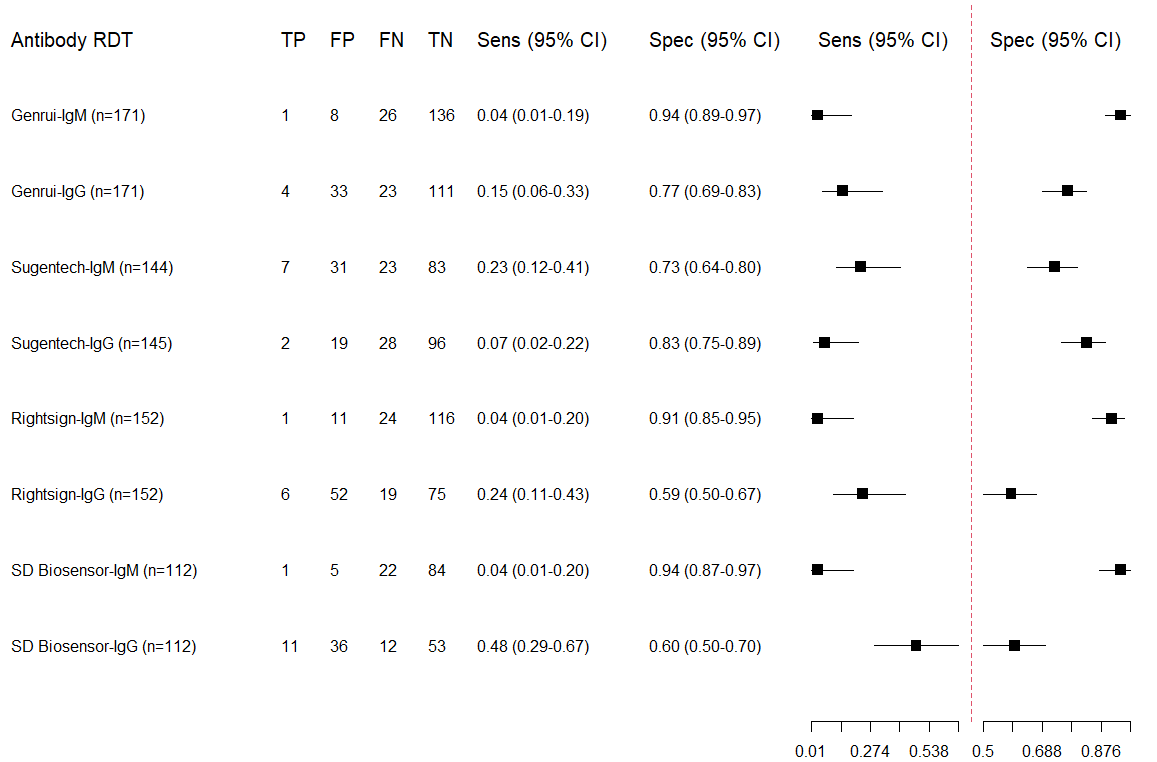


TP – True positive; FP - False positive; FN - False negative, TN - True negative; Sens-Sensitivity; Spec-Specificity
