# Supplementary material for: Performance evaluation of SARS-CoV-2 rapid diagnostic tests in Nigeria: A cross-sectional study
Source: PLOS Glob Public Health. 2024 Jul 15;4(7):e0003371. doi: 10.1371/journal.pgph.0003371 (PMC11249252; doi:10.1371/journal.pgph.0003371)
Supplement: S2 Checklist — (DOCX) [file pgph.0003371.s004.docx]

STROBE Statement—checklist of items that should be included in reports of observational studies

|  | Item No. | Recommendation | Page  No. | Relevant text from manuscript |
| --- | --- | --- | --- | --- |
| **Title and abstract** | 1 | (*a*) Indicate the study’s design with a commonly used term in the title or the abstract | 1 | Performance Evaluation of SARS-CoV-2 Rapid Diagnostic Tests in Nigeria: A Cross-sectional Study |
|  |  | (*b*) Provide in the abstract an informative and balanced summary of what was done and what was found | 4 | This study assessed the sensitivity, specificity, and predictive values of ten rapid diagnostic tests (RDTs) kits for COVID-19 testing in Nigeria.  Ag-RDTs had better performance characteristics compared with Ab-RDTs; however, the sensitivities of all RDTs evaluated in this study were generally low. The relatively high specificity of Ag-RDTs makes them useful for the diagnosis of infection in COVID-19 suspected cases where positive RDT may not require confirmation by molecular testing. |
| Introduction | | | |  |
| Background/rationale | 2 | Explain the scientific background and rationale for the investigation being reported | 6 - 7 | Rapid diagnostic tests (RDTs), such as antibody (Ab) and antigen (Ag) RDTs, are attractive alternatives to the technically demanding, resource intensive, though highly sensitive molecular platforms in remote settings in Africa. However, the performance characteristics of commercially available kits are yet to be evaluated in a nationally representative field study to ascertain their reliability. |
| Objectives | 3 | State specific objectives, including any prespecified hypotheses | 7 | This study was conducted to assess the sensitivity, specificity, and predictive values of RDTs available for point-of-care COVID-19 diagnosis in Nigeria. |
| Methods | | | |  |
| Study design | 4 | Present key elements of study design early in the paper | 7 | This multi-centre cross-sectional study was conducted in thirteen (13) states and the Federal Capital Territory (FCT), within the six (6) geo-political zones of Nigeria |
| Setting | 5 | Describe the setting, locations, and relevant dates, including periods of recruitment, exposure, follow-up, and data collection | 7 | The study was carried out for four months (October 2021 to January 2022) and spanned through two successive waves (3rd and 4th) of the COVID-19 pandemic. Government-approved testing centres were used to collect samples from consenting participants suspected of COVID-19 in 13 states of the Country. |
| Participants | 6 | *Cross-sectional study*—Give the eligibility criteria, and the sources and methods of selection of participants | 11 | Consecutive consenting adults (18 years and above) who presented at the various centres for COVID-19 testing were enrolled in the study for four months (October 2021 to January 2022). |
|  |  |  |  |  |
| Variables | 7 | Clearly define all outcomes, exposures, predictors, potential confounders, and effect modifiers. Give diagnostic criteria, if applicable | 11 – 12  14  14  14 | Concomitant nasopharyngeal, oropharyngeal, and nasal swabs as well as whole blood samples, were collected from consenting individuals attending the various sample collection centres for COVID-19 testing.  Socio-demographic information obtained from participants, and the laboratory results (RT-PCR and RDTs results) were entered into the research electronic data capture (REDCap)    Percentage concordance based on agreed criteria, sensitivity, specificity, positive predictive values, negative predictive values, and the area under the receiver operating characteristic curve was used to determine the general performance and characteristics of the RDTs.  The agreed criteria for percentage concordance were set at 95% sensitivity (positive concordance) and 90% specificity (negative concordance) when compared to the RT-PCR gold standard. |
| Data sources/ measurement | 8* | For each variable of interest, give sources of data and details of methods of assessment (measurement). Describe comparability of assessment methods if there is more than one group |  | Socio-demographic information - participant administered questionnaire, and  Laboratory results – collated from the different study centres |
| Bias | 9 | Describe any efforts to address potential sources of bias |  | We minimised selection bias by using a single and standard eligibility criteria across the study sites. |
| Study size | 10 | Explain how the study size was arrived at | 11 and 28  n =[ Z_α_ √ 2 X P⁻ (1 - P⁻) + Z_β_√P_1_ (1- P_1_) + P_2_ (1- P_2)_]^2^  (P_1_ – P_2_ )^2^ | Hajian-Tilaki, K. Sample size estimation in diagnostic test studies of biomedical informatics. J. Biomed Inform. 2014;48:193-204. doi: 10.1016/j.jbi.2014.02.013. Epub 2014 Feb 26 |

Continued on next page

| Quantitative variables | 11 | Explain how quantitative variables were handled in the analyses. If applicable, describe which groupings were chosen and why | 14 | All data were coded and entered into REDCap by individual study centres. The pooled data was analysed centrally. |
| --- | --- | --- | --- | --- |
| Statistical methods | 12 | (*a*) Describe all statistical methods, including those used to control for confounding | 14 | Statistical analysis was done using StataCorp. 2019. Stata Statistical Software: Release 16. College Station, TX: StataCorp LLC. Descriptive statistics (percentages and frequencies) were used for the socio-demographic data. The area under the receiver operating characteristic curve (AUROC) was computed for individual test kits using the sensitivity and 1-specificity of each test to plot a curve across varying cut-off values |
|  |  | (*b*) Describe any methods used to examine subgroups and interactions |  | All data obtained were pooled and analysed. Sub-group analysis was not done |
|  |  | (*c*) Explain how missing data were addressed | 14 | We recorded a small number of missing data for covariates and so we adopted the ‘missing indicator’ approach (coded as “no response”) |
|  |  | *Cross-sectional study*—If applicable, describe analytical methods taking account of sampling strategy | 14 | We used a combination of descriptive analysis and diagnostic accuracy analysis; for the latter we modified an existing do-file in Stata to compute area under the receiver operating characteristic curve (AUROC), sensitivity, specificity, positive predictive value and negative predictive value with their respective 95% CI. |
|  |  | (*e*) Describe any sensitivity analyses |  | Not applicable |
| Results | | | | |
| Participants | 13* | (a) Report numbers of individuals at each stage of study—eg numbers potentially eligible, examined for eligibility, confirmed eligible, included in the study, completing follow-up, and analysed | 15 | One thousand, three hundred and ten (1,310) respondents comprising 767 (58.5%) males and 543 (41.5%) females were confirmed eligible and enrolled in the study |
|  |  | (b) Give reasons for non-participation at each stage | 15 | Samples were collected from all consenting participants. Individuals who did give consent were excluded |
|  |  | (c) Consider use of a flow diagram |  | Not applicable |
| Descriptive data | 14* | (a) Give characteristics of study participants (eg demographic, clinical, social) and information on exposures and potential confounders | 15 | The highest proportion of participants, 757 (57.7%), were in the 20-39 years age group. Seven hundred and twenty-nine (55.6%) participants were married and 654 (49.9%) practised Islam. The Hausa ethnic group had the highest frequency (547; 41.8%) while 347 (26.5%) were unskilled labourers, and 767 (58.5%) had higher than secondary school education. |
|  |  | (b) Indicate number of participants with missing data for each variable of interest | 15 | Please see Table 2 for details of participants’ characteristics with missing records (i.e., No response). However, missing data is not applicable to the diagnostic tests—the primary focus of this study. |
|  |  |  |  |  |
| Outcome data | 15* |  |  |  |
|  |  |  |  |  |
|  |  | *Cross-sectional study—*Report numbers of outcome events or summary measures | 19-21 | AUROC, sensitivity, specificity, positive predictive value, and negative predictive value. |
| Main results | 16 | (*a*) Give unadjusted estimates and, if applicable, confounder-adjusted estimates and their precision (eg, 95% confidence interval). Make clear which confounders were adjusted for and why they were included |  | Not applicable |
|  |  | (*b*) Report category boundaries when continuous variables were categorized |  | Not applicable |
|  |  | (*c*) If relevant, consider translating estimates of relative risk into absolute risk for a meaningful time period |  | Not applicable |

Continued on next page

| Other analyses | 17 | Report other analyses done—eg analyses of subgroups and interactions, and sensitivity analyses |  | All analyses are shown in the manuscript |
| --- | --- | --- | --- | --- |
| Discussion | | | | |
| Key results | 18 | Summarise key results with reference to study objectives | 21 - 22 | Ag-RDTs evaluated in this study appeared to perform better than antibody-based tests in the diagnosis of COVID-19. Although all Ag-RDTs showed good specificity with most exceeding 97%, their sensitivities were far below the benchmark of at least 80% set by WHO |
| Limitations | 19 | Discuss limitations of the study, taking into account sources of potential bias or imprecision. Discuss both direction and magnitude of any potential bias | 24 | The study had a few limitations, which include: (1) a comprehensive head-to-head comparison of the Ag-RDTs was not feasible because most enrollees did not consent to the collection of multiple swabs; (2) comparability of the results may be limited by the non-uniformity in SARS-CoV-2 assays employed for molecular diagnosis at the various sites; and (3) RT-PCR tests, despite being the gold standard, is known to detect a non-viable viral nucleic acid. This may result in an erroneous perception of low performance in the RDTs. |
| Interpretation | 20 | Give a cautious overall interpretation of results considering objectives, limitations, multiplicity of analyses, results from similar studies, and other relevant evidence | 24 | Antibody-based RDTs may have limited use in detecting acute disease but they may be useful in the surveillance of recent and past COVID-19 infections, this however needs to be evaluated. Ag-RDTs may be used to quickly detect COVID-19 at reduced cost in clinical settings but negative results must be interpreted cautiously and supplemented with additional PCR testing where clinical suspicion is high. |
| Generalisability | 21 | Discuss the generalisability (external validity) of the study results | 24 | The RDTs evaluated are not sensitive enough to replace RT-PCR for the diagnosis of COVID-19. However, the high specificity of Ag-RDTs makes them useful supplements capable of reducing the need for molecular testing in confirming COVID-19 positive samples in Nigeria. |
| Other information | |  | | |
| Funding | 22 | Give the source of funding and the role of the funders for the present study and, if applicable, for the original study on which the present article is based | 25 | The CORVAN study was coordinated by the Nigerian COVID-19 Research Coalition (NCRC) with funding support from the World Bank through a grants to Regional Disease Surveillance Systems Enhancement (REDISSE). |

*Give information separately for cases and controls in case-control studies and, if applicable, for exposed and unexposed groups in cohort and cross-sectional studies.

**Note:** An Explanation and Elaboration article discusses each checklist item and gives methodological background and published examples of transparent reporting. The STROBE checklist is best used in conjunction with this article (freely available on the Web sites of PLoS Medicine at http://www.plosmedicine.org/, Annals of Internal Medicine at http://www.annals.org/, and Epidemiology at http://www.epidem.com/). Information on the STROBE Initiative is available at www.strobe-statement.org.
